# Acute-phase clinical profile and quality of life in patients with chikungunya during the 2025 outbreak in Bangladesh: A facility-based cross-sectional study

**DOI:** 10.1371/journal.pntd.0014599

**Published:** 2026-08-26

**Authors:** Mohammad Jahid Hasan, Md. Shahabul Huda Chowdhury, Muna Islam, Sadia Islam, Tanzin Naher, H.M. Hamidullah Mehedi, Abu Saif Mohammad Lutful Kabir, Jannatul Fardous, Refaya Tasnim, Asif Ahmed, Md. Atiquel Islam Chowdhury, Madhabi Karmaker, Md. Mahir Faisal Akash, Mohiuddin Sharif, Quazi Tarikul Islam

**Affiliations:** 1 Department of Public Health, Tropical Disease and Health Research Center, Dhaka, Bangladesh; 2 Department of Medicine, Dhaka Medical College and Hospital, Dhaka, Bangladesh; 3 Department of Medicine, Chittagong Medical College Hospital, Chattogram, Bangladesh; 4 Department of Medicine, Delta Medical College, Dhaka, Bangladesh; 5 Laboratory Medicine, Bangladesh Medical University, Dhaka, Bangladesh; 6 Department of Medicine, 250 Bedded General Hospital, Chattogram, Bangladesh; 7 Department of Psychiatry, National Institute of Mental Health (NIMH), Dhaka, Bangladesh; 8 Department of Medicine, BRB Hospitals Limited, Dhaka, Bangladesh; 9 Department of Medicine, Popular Medical College Hospital, Dhaka, Bangladesh; 10 Department of Medicine, Southern Medical College, Chattogram, Bangladesh; 11 Department of Public Health, Pi Research and Development Centre, Dhaka, Bangladesh; Central University of Tamil Nadu, INDIA

## Abstract

**Background:**

Bangladesh experienced a notable resurgence of chikungunya cases in 2025, following its last major outbreak in 2017. This study aimed to characterize the epidemiological profile of laboratory-confirmed chikungunya cases in Bangladesh.

**Methods:**

This cross-sectional study included and interviewed 442 laboratory-confirmed adult chikungunya cases. Pain was assessed using the Visual Analog Scale (VAS, 0–10) and quality of life was measured via the validated Bangla EQ-5D-3 L instrument, which evaluates five domains—mobility, self-care, usual activities, pain/discomfort, and anxiety/depression. Overall health was rated using the EQ-VAS, a vertical scale ranging from 0 (“worst imaginable health state”) to 100 (“best imaginable health state”). Index scores were derived using the trade-off (TTO) valuation set. Associations with the overall EQ-5D index were analyzed using Tobit regression, whereas domain-specific associations were examined via ordinal logistic regression models.

**Results:**

Of all, 16.5% of patients required hospital admission, with a median length of stay of 5 days (IQR 3–6), and the rest of them consulted on an outdoor basis. The mean (SD) age of the participants was 45·3 (15·5) years, and 60% were female. Fever (98%) and arthralgia (97%) were the predominant symptoms. Mild pain was reported by 6% of patients, while 40% had moderate and 54% had severe pain, with a median VAS score of 8.0 (IQR 7.0–9.0). Over 70% of the participants reported mobility limitations, self-care, functional impairment, pain or psychological symptoms. The median EQ-5D index was 0·78 (IQR 0·60-0·96). Female sex (β = –0·10, 95% CI –0·17, –0·02) and greater pain severity (β = –0·26, 95% CI –0·37, –0·15) were independently associated with a lower EQ-5D index.

**Conclusions:**

During the outbreak, chikungunya patients in Bangladesh predominantly presented with fever and joint pain and experienced marked declines in quality of life, with women being disproportionately affected.

## Author summary

In 2025, Bangladesh experienced a renewed outbreak of chikungunya after its last major epidemic in 2017. We assessed 442 adults with laboratory-confirmed chikungunya to describe their clinical features and the impact of the illness on daily functioning and well-being. Nearly all patients reported fever and joint pain, and more than half experienced severe pain. A large proportion of participants reported difficulties with walking, self-care, usual activities, or emotional well-being, indicating a substantial burden of illness. Although most patients were managed as outpatients, approximately one in six required hospital admission. Overall perceived health and quality of life were markedly reduced during the acute phase of infection. Women and individuals with more intense pain experienced disproportionately greater impairment. These findings highlight the considerable individual and health system burden of chikungunya during outbreaks and underscore the need for effective clinical management and supportive care strategies in endemic settings.

## Introduction

Chikungunya is a mosquito-borne viral disease transmitted to humans primarily through the bites of *Aedes aegypti* and *Aedes albopictus* mosquitoes [1]. First identified in Tanzania in 1952, chikungunya has since established endemic or epidemic transmission in more than 110 countries across tropical and subtropical regions [2,3]. Globally, an estimated 35 million infections occur each year, with the highest burden reported from South and Southeast Asia, sub-Saharan Africa, and the Americas [3].

In Bangladesh, chikungunya was first detected in 2008 [4], followed by the country’s largest outbreak in 2017, which resulted in more than 13,000 clinically confirmed cases nationwide [5–7]. After several years of minimal transmission, surveillance by the Institute of Epidemiology, Disease Control and Research (IEDCR) identified 337 cases between January and May 2025, including 153 confirmed by PCR [8]. Subsequently, the World Health Organization (WHO) reported 732 suspected and 400 laboratory-confirmed cases in Dhaka by August 2025 [9]. This resurgence indicates a re-emergent transmission pattern and raises concern for a large-scale national outbreak [4].

Clinically, chikungunya is characterized by the sudden onset of high-grade fever, severe polyarthralgia, headache, myalgia, and nausea, often accompanied by rash and fatigue [2,10]. These manifestations typically appear after an incubation period of two to seven days and can cause substantial level of morbidity despite the generally self-limiting nature of the infection [10]. In Bangladesh, similar patterns of presentation were reported during earlier outbreaks [6,7,11–13]. However, re-emergence of chikungunya after a quiescent period warrants a systematic investigation of the clinical presentations and laboratory findings in these patients considering the fact that clinical expression and severity may vary across outbreaks due to viral strain differences, host susceptibility, or environmental factors [4,14–16]. Moreover, in the context of co-circulating arboviral infections such as dengue, distinguishing chikungunya based on its clinical and laboratory profile remains a diagnostic challenge, further emphasizing the need for updated epidemiological and clinical characterization [4].

Although chikungunya-associated mortality remains uncommon, the disease is known for causing significant physical disability and impaired quality of life of the patients. Approximately half of the patients develop chronic disability, lasting months to years after the acute phase of infection [2]. However, quality of life impairment is not limited to the chronic stage; substantial deterioration occurs during the acute illness as well. A previous study from Bangladesh during the 2017 outbreak reported that approximately 83% of affected individuals experienced moderate to severe impairment in quality of life [7], whereas such data remain limited for the 2025 outbreak [16].

In this context, the objective of our study was to investigate the clinical presentation, laboratory profile, and quality of life during acute-phase infection among patients with chikungunya during the 2025 outbreak in Bangladesh.

## Methods

### Ethics statement

The study adhered to the principles of the current Declaration of Helsinki 2024. The study protocol was reviewed and approved by the Institutional Review Board of Public Health Foundation, Bangladesh (Ref: PHFBD-ERC-NFP-R-28/2025). All participants provided written informed consent after receiving an explanation of study objectives, procedures, and potential risks. Participation was voluntary, and respondents could withdraw at any stage without affecting their medical care. Data were anonymized using unique participant codes, and all personal identifiers were removed prior to analysis.

### Study design and participants

We conducted a facility-based cross-sectional study in Dhaka Medical College Hospital, Chittagong Medical College Hospital, Chattogram, 250 bedded General Hospital, Chattogram, Popular Medical College Hospital, Dhaka and Delta Medical College Hospital, Dhaka between August and October 2025.

The study population comprised adult patients (age ≥ 18 years) diagnosed with chikungunya infection according to the Bangladesh National Guideline on Clinical Management of Chikungunya Fever. The case definition for chikungunya was the presence of acute onset fever (≥38.5°C) accompanied by severe arthralgia or arthritis unexplained by other medical conditions, along with an epidemiological link to a confirmed outbreak area and laboratory confirmation by either the detection of chikungunya-specific IgM antibodies using enzyme-linked immunosorbent assay (ELISA) or the identification of chikungunya virus RNA in serum samples through reverse transcriptase polymerase chain reaction (RT-PCR) [14,17,18]. However, patients were admitted to hospital according to the clinical judgment and discretion of the attending emergency physicians, based on severity of symptoms and need for inpatient care.

We calculated sample size for this study from the following formula: n = z^2^p(1-p)/d^2^, where, z was considered as 1.96 for 95% confidence level, p was proportion of patients with chikungunya who reported impaired quality of life, considered as 50% (as there is no previous data in our country), and d was precision of error, considered as 0.05. From these assumptions, the calculated sample size was 385. Considering 15% non-response, the total estimated sample size was 442. In this study, a total of 442 RT-PCR confirmed case were included in this study by convenience sampling. Patients were excluded if they were pregnant, had mixed infections with dengue or other febrile illnesses confirmed by laboratory testing, or were unable to provide information due to altered sensorium or critical illness.

### Data collection

After confirming eligibility and obtaining written informed consent, the attending physician conducted a face-to-face interview with each participant within 48 hours of admission using a pretested case record form (CRF). The CRF included sections on sociodemographic information, clinical presentations, laboratory parameters, and quality-of-life assessment. Interviews were conducted in Bangla in a private clinical setting to ensure comfort and confidentiality.

Clinical data included onset, duration, and pattern of fever, arthralgia, rash, myalgia, headache, conjunctivitis, gastrointestinal symptoms, and neurological features. Joint involvement was categorized according to the anatomical sites affected (e.g., small joints, wrists, elbows, knees, and ankles etc.). The severity of pain was evaluated using the Visual Analog Scale (VAS, 0–10). For clinical interpretation, VAS scores were further categorized as mild (0-3.9), moderate (4-6.9), and severe (7–10) pain [19].

Laboratory tests included serological confirmation of chikungunya virus, molecular detection of viral RNA in early illness, screening for co-infections, and routine hematological and biochemical investigations. Venous blood samples were collected under aseptic conditions at the time of patient presentation. Serological testing was performed using a commercially available ELISA kit for anti-chikungunya IgM antibodies (Novatec Chikungunya IgM Capture ELISA kits, NovaTec Immundiagnostica GmbH, Germany) following the manufacturer’s instructions. Samples collected within the first five days of illness were additionally tested by real-time reverse transcription polymerase chain reaction (RT-PCR) for chikungunya viral RNA using a commercially available multiplex real-time RT-PCR assay for chikungunya, dengue, and Zika viruses (Advanced Molecular Diagnostics Ltd, UK), performed according to the manufacturer’s instructions [14]. To exclude co-infections with other arboviruses like dengue, all samples were concurrently screened for dengue virus NS1 antigen and dengue IgM antibodies using ELISA kits (DENV Detect NS1 ELISA Kit and DENV Detect IgM ELISA Kit, InBios International, Inc., USA). Routine laboratory investigations included complete blood count, liver function tests, and renal function tests.

Health-related quality of life (QoL) was assessed using the Bangla version of the EuroQol five-dimension three-level questionnaire (EQ-5D-3L) [20]. The EQ-5D-3L comprises five domains, such as mobility, self-care, usual activities, pain/discomfort, and anxiety/depression, each with three response levels: no problems, some problems, and extreme problems/unable to perform. Participants self-reported their health status for each domain on the day of assessment, reflecting their acute-phase condition, and responses were coded as ordinal variables (1–3), with higher scores indicating greater impairment. In addition, overall health was rated using the EQ visual analogue scale (EQ-VAS), a 20-cm vertical scale ranging from 0 (“worst imaginable health state”) to 100 (“best imaginable health state”) [21], providing a continuous measure of self-perceived health during the acute phase of infection.

### Statistical analysis

All completed CRFs were cross-checked daily for accuracy and completeness and then entered into a password-protected database. Continuous variables were first assessed for normality using visual inspection of histograms. Depending on the distribution, continuous variables were summarized either as mean with standard deviation (SD) for or as median with interquartile range (IQR). Categorical variables were presented as frequencies with percentages.

For quality-of-life data, we reported the proportion of patients reporting each level (no problems, some problems, extreme problems) within each EQ-5D-3L domain. Subsequently, we calculated the EQ-5D index score using the time trade-off (TTO) valuation set established for the general population of Pakistan, another lower middle income country of south Asia [22], in the absence of a locally validated set for Bangladesh. This index provided a societal preference-weighted measure of health utility, scaled from 0 (equivalent to death) to 1 (representing perfect health). Considering the bounded nature of the EQ-5D index (0–1) and the presence of both left-censoring at 0 and right-censoring at 1, we employed Tobit regression models to examine associations between patient characteristics and overall QoL [23]. Tobit regression is well-suited for dependent variables that are censored at known thresholds, allowing unbiased estimation of regression coefficients and accounting for latent health utility scores that extend beyond observed bounds [24]. In our models, the EQ-5D index served as the dependent variable, while age (continuous, per year increase), sex (female vs male), pain severity (ordinal: mild, moderate, severe), and comorbidity status (any vs none) were included as predictors. Laboratory parameters including the inflammatory biomarkers were not included in multivariable analyses due to substantial missingness of data across variables, with available sample sizes ranging from 42 to 88 in laboratory-specific variables. Inclusion of these variables would have resulted in a reduction in effective sample size and increased risk of unstable estimates due to incomplete case analysis. Therefore, to preserve model stability and maximize statistical power, analyses were restricted to clinical variables that were available for the majority of participants. Model fitness was tested using residual scale, log-likelihood, and overall model significance statistics. For domain-specific analyses, we used ordinal logistic regression (proportional odds models) to account for the ordered nature of the responses [23]. Each EQ-5D-3L domain was modeled separately, with the same set of predictors. The proportional odds assumption was evaluated by comparing cumulative logit coefficients across response thresholds, and no significant violations were observed. Coefficients were exponentiated to generate odds ratios (ORs) with 95% confidence intervals (CI), representing the odds of reporting a higher level of severity relative to the reference category.

As supplementary, bivariable analyses were performed to examine associations between laboratory parameters (IgG, IgM, RT-PCR status, platelet count, and liver enzymes) and outcomes including pain severity and EQ-5D index, using chi-square or Fisher’s exact tests for categorical variables and independent sample t-tests for continuous outcomes.

The statistical significance threshold was set at two-sided p < 0.05, and all analyses were conducted using R version 4.4.2.

## Results

### Participants’ characteristics

A total of 442 laboratory-confirmed chikungunya cases were included in this study. Their mean age was 45.3 years (SD 15.5), and 60% were female. Hypertension and type 2 diabetes mellitus were most common comorbidities, reported in 24% and 17% of the patients, respectively, followed by dyslipidemia (10%), coronary artery disease (4%), and hypothyroidism (3%). Around 73% were positive for IgM antibody and 27% for RT-PCR. A total of 16.5% of patients required hospital admission with a median length of stay of 5 days (IQR 3–6) (Table 1).

**Table 1:** Sociodemographic, clinical and laboratory profile of the patients with chikungunya (n = 442).

Characteristics	Values
Age (years), mean (SD)	45.3 (15.5)
Sex, n (%)	
Male	177 (40.1)
Female	264 (59.9)
Comorbidities, n (%)	
Type 2 diabetes mellitus	76 (17.2)
Hypertension	107 (24.2)
Coronary artery disease	18 (4.1)
Dyslipidemia	43 (9.7)
Hypothyroidism	12 (2.7)
Serological tests, n (%)	
IgM positive	323 (73.1)
PCR positive	119 (26.9)
**Clinical presentation, n (%)**	
Fever	433 (97.9)
Duration of fever (days), median (IQR)	5.0 (3.0-7.0)
Highest recorded temperature (^o^c), median (IQR)	39.4 (38.9-40.0)
Joint pain	430 (97.3)
Severity of pain (n = 430)	
Mild	27 (6.3)
Moderate	172 (39.9)
Severe	232 (53.8)
VAS score for severity of pain, median (IQR) (n = 430)	8.0 (7.0-9.0)
Joint swelling	230 (52.0)
Joint tenderness	31 (7.0)
Involved joint (n = 430)	
Knee	263 (59.5)
Elbow	128 (28.9)
Shoulder	111 (25.1)
Ankle	247 (55.9)
Foot	165 (37.3)
Wrist	43 (9.7)
Hip	8 (1.8)
Back and neck	25 (5.6)
Small joints	206 (46.6)
Headache	33 (7.5)
Retro-orbital pain	58 (13.1)
Conjunctivitis	16 (3.6)
Myalgia	262 (59.3)
Rash	236 (53.4)
Mouth ulcer	54 (12.2)
Nausea	184 (41.6)
Vomiting	151 (34.2)
Diarrhea	65 (14.7)
Abdominal pain	43 (9.7)
Abdominal distention	26 (5.9)
**Laboratory parameters, median (IQR)**	
Hemoglobin (g/dL) (n = 88)	11.8 (11.2-12.8)
Hematocrit (%) (n = 85)	36.7 (34.3-39.4)
ESR (mm/hour) (n = 78)	28.5 (12.3-40.0)
WBC (×10³/µL) (n = 88)	7.0 (4.9-8.8)
Platelet (×10^5^/µL) (n = 87)	2.2 (1.6-2.8)
RBS (mmol/L) (n = 53)	6.1 (5.0-8.3)
ALT (U/L) (n = 73)	30.0 (20.5-39.0)
AST (U/L) (n = 51)	30.0 (20.0-40.0)
Serum creatinine (mg/dL) (n = 57)	0.9 (0.7-1.1)
Serum uric acid (mg/dL) (n = 42)	5.2 (4.1-6.5)
Hospital admission required, n (%)	73 (16.52)
Duration of hospital stay (days), median (IQR) (n = 73)	5.0 (3.0-6.0)

### Clinical presentations

Fever was present in nearly all the patients (98%), with a median duration of 5 days (IQR 3–7) and a peak temperature of 39.4°C (IQR 38.9-40.0). Arthralgia was reported by 97% of patients, with pain categorized as mild in 6%, moderate in 40%, and severe in 54%; the median VAS score for pain was 8.0 (IQR 7.0-9.0). Joint swelling and tenderness were present in 52% and 7% of the patients, respectively, predominantly affecting knees, ankles, and small joints. Other frequent symptoms included myalgia (59%), rash (53%), nausea (42%), vomiting (34%), retro-orbital pain (13%), and headache (7.5%) (Table 1). No severe systemic complications, such as neurological or cardiac manifestations, were observed among the participants during the study period.

### Laboratory parameters

Hematological indices of the patients were largely within normal ranges, with median hemoglobin of 11.8 g/dL and hematocrit of 36.7%, while white blood cell counts and platelet counts had median values of 7.0 × 10³/µL and 2.2 × 10^5^/µL, respectively. Median erythrocyte sedimentation rate was 28.5 mm/hour, median random blood glucose was 6.1 mmol/L. Median of liver enzyme levels (ALT and AST) was 30 U/L, median of serum creatinine was 0.9 mg/dL, and median of serum uric acid was 5.2 mg/dL (Table 1). Thrombocytopenia and transaminitis were uncommon, with two patients showing platelet counts <100,000/µL, and mild elevations of ALT (>48 U/L) and AST (>48 U/L) observed in 11 and 3 patients, respectively.

### Quality of life

In the mobility domain, 63% of patients reported some problems and 22% were confined to bed. Self-care and usual activities were affected in 53% and 58% of patients, respectively, with 18% and 21% unable to perform basic tasks. Pain and discomfort were common, with 58% reporting moderate pain and 21% extreme pain. Anxiety or depression was reported by 51% as moderate and 11% as severe. Median EQ-VAS score was 50 (IQR 30–70), and EQ-5D index was 0.78 (IQR 0.60-0.96) (Table 2).

**Table 2:** Health-related quality of life of the patients with chikungunya (n = 442).

Domains	n (%) or Median (IQR)
Mobility	
No problems	67 (15.2)
Some problems	280 (63.4)
Confined to bed	95 (21.5)
Self-care	
No problems	126 (28.5)
Some problems	236 (53.4)
Unable to wash/dress	80 (18.1)
Usual Activities	
No problems	94 (21.3)
Some problems	257 (58.1)
Unable to perform	91 (20.6)
Pain/ Discomfort	
No pain	94 (21.3)
Moderate pain	256 (57.9)
Extreme pain	92 (20.8)
Anxiety/ Depression	
Not anxious/depressed	170 (38.5)
Moderately anxious/depressed	223 (50.5)
Extremely anxious/depressed	49 (11.1)
VAS (0–100), median (IQR)	50 (30-70)
EQ-5D Index (0–1), median (IQR)	0.78 (0.60-0.96)

### Factors associated with quality of life

In Tobit regression model, female sex was significantly associated with lower EQ-5D index compared with males (β = –0.10, 95% CI –0.17 to –0.02; p = 0.010). Pain severity showed the largest negative effect, with each level increase (from mild to moderate to severe) corresponding to a marked decline in EQ-5D index (β = –0.26, 95% CI –0.37 to –0.15; p < 0.001). However, increasing age and comorbidity was not significantly associated with EQ-5D index (β = –0.02, 95% CI –0.03 to 0.01; p = 0.066 for age and β = –0.01, 95% CI –0.09 to 0.07; p = 0.940 for any comorbidity) (Table 3). Model fit was evaluated using the log-likelihood, which was –221.8. The overall model was statistically significant (Wald χ² = 56.53, df = 5, p < 0.001), indicating that the set of predictors collectively explained variation in the EQ-5D index. The residual variability of the latent EQ-5D index was represented by the scale parameter, with a standard deviation of 0.3645, reflecting acceptable model fit.

**Table 3:** Factors associated with overall EQ-5D index (Tobit regression model).

Variable	β (95% CI)	P-value
Age (per year increase)	–0.02 (–0.03, 0.01)	0.066
Female (vs Male)	–0.10 (–0.17, –0.02)	0.010
Pain severity (linear)	–0.26 (–0.37, –0.15)	<0.001
Comorbidity (any vs none)	–0.01 (–0.09, 0.07)	0.940

**Fig 1:**
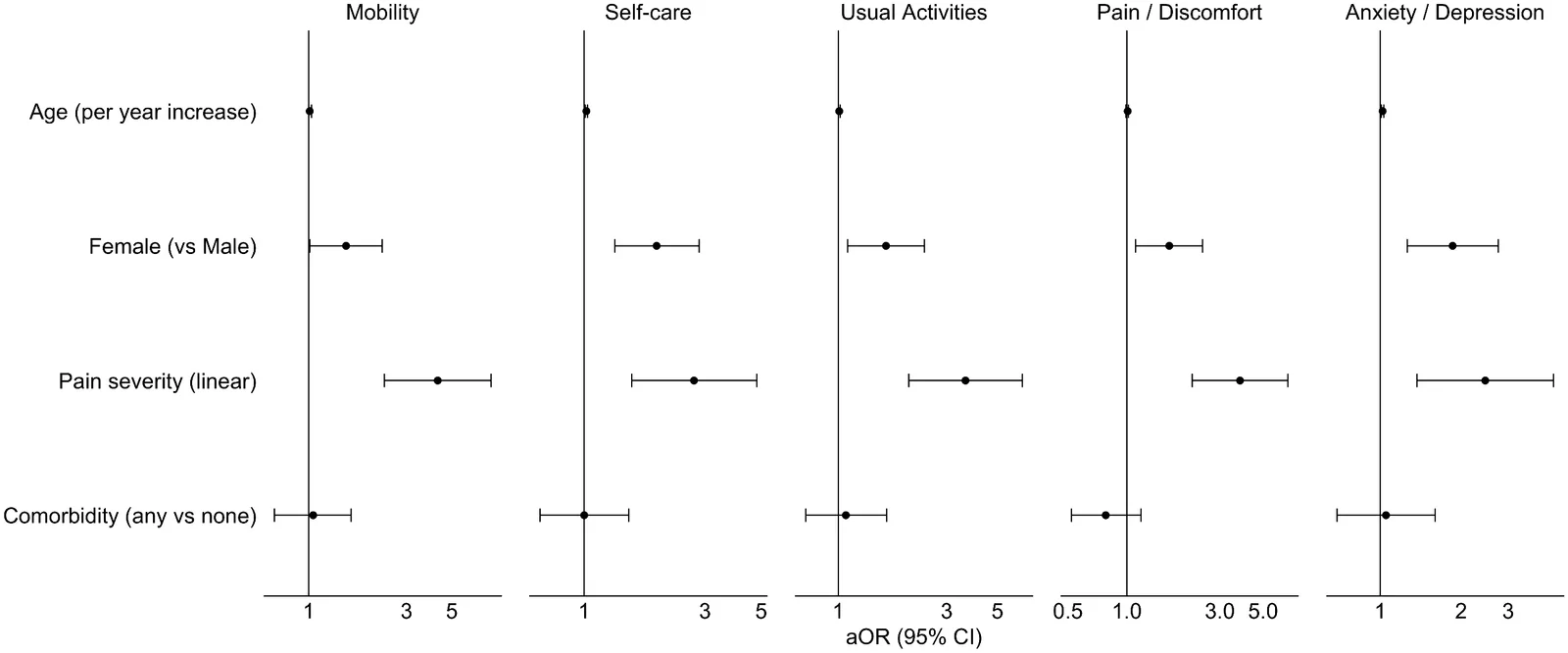
Factors associated with domains of quality of life in patients with chikungunya (ordinal logistic regression model).

In ordinal logistic regression analyses for the factors associated with specific domains of EQ-5D, female sex was consistently associated with higher odds of reporting problems across all domains, including mobility (aOR 1·52, 95% CI 1·01, 2·28), self-care (aOR 1·93, 95% CI 1·32, 2·84), usual activities (aOR 1·62, 95% CI 1·10, 2·39), pain/discomfort (aOR 1·65, 95% CI 1·11, 2·44), and anxiety/depression (aOR 1·86, 95% CI 1·26, 2·75). Similarly, pain severity was associated with higher odds of reporting problems across all EQ-5D domains, including mobility (aOR 4·26, 95% CI 2·34, 7·76), self-care (aOR 2·71, 95% CI 1·54, 4·79), usual activities (aOR 3·62, 95% CI 2·04, 6·43), pain/discomfort (aOR 3·80, 95% CI 2·16, 6·69), and anxiety/depression (aOR 2·46, 95% CI 1·37, 4·42). However, age and presence of comorbidities were not significantly associated with any domain (Fig 1).

IgG seropositivity was significantly associated with pain severity (p < 0.001), with a higher proportion of milder pain categories observed among IgG-positive patients. No significant associations were observed for IgM, PCR, platelet count, or liver enzyme abnormalities (S1 Table). No statistically significant differences in EQ-5D index scores were observed across categories of IgG, IgM, PCR, platelet count, or liver enzymes. A non-significant trend toward higher EQ-5D scores was observed among IgG-positive and PCR-positive patients (S2 Table).

## Discussion

Among our included 442 patients with chikungunya, the majority presented with fever, severe arthralgia, and rash, with nearly one in six requiring hospital admission. Biochemical and hematological parameters remained within reference ranges in majority of the patients. Quality of life was impaired across all EQ-5D domains during the acute phase of infection, particularly in mobility, pain/discomfort, and psychological wellbeing. Female sex and greater pain severity were independently associated with lower EQ-5D index scores and higher odds of reporting functional limitations across all health domains, whereas age and comorbidity showed no significant association.

The clinical presentation observed in our study during 2025 outbreak closely aligns with reports from earlier chikungunya outbreaks in Bangladesh [6,7,11–13,16] as well as in other countries of South and Southeast Asia like India [25], Pakistan [26], Sri Lanka [27], Indonesia [28], Maldives [29], and Thailand [30] etc., where fever and polyarthralgia remain most common features followed by joint swelling, rash, headache, myalgia etc. This consistency across settings likely reflects the conserved clinical manifestations of chikungunya virus infection across different lineages, where joint tropism and inflammatory responses remain dominant pathophysiological features. Similar to our findings, several previous studies reported joint involvement in chikungunya, symmetrically affecting both large and small joints, particularly the knees, ankles, and small hand joints [12,16]. Besides, the hematological and biochemical parameters remained mostly within normal range in our study, similar to findings of previous studies in Bangladesh, where around 10% pf the patients reported alteration in these parameters [12,13]. The relatively limited hematological change may be explained by the fact that chikungunya primarily induces immune-mediated inflammatory responses rather than direct hematopoietic suppression, unlike dengue where plasma leakage and cytopenias are more prominent.

Chikungunya exerts a significant adverse effect on health-related quality of life of the patients [2,31,32]. Previous studies have consistently demonstrated that higher pain intensity among affected individuals is strongly associated with reduced physical functioning and impaired mental well-being [32–34]. In our study, more than 80% of patients reported limitations in mobility, inability to perform usual activities, or moderate-to-severe pain, while over half experienced moderate-to-severe psychological distress, including anxiety or depression, as measured by the EQ-5D scale. The magnitude of impairment observed in our study appears comparable to findings from previous studies, suggesting that acute chikungunya has a consistently high disability burden across endemic settings [2,31,32]. The strong association between pain severity and impaired quality of life observed in our findings also aligns with earlier studies [7,32–34]. This association is biologically plausible, as chikungunya-related arthralgia is driven by intense inflammatory cytokine activation, which directly contributes to functional restriction and psychological distress through persistent pain signaling. Furthermore, the association between female sex and lower EQ-5D index scores, as well as greater odds of impairment across all domains, indicates potential gender-related vulnerabilities. These may reflect biological differences in pain perception and gender-specific psychosocial stressors [35,36]. Besides, differences in healthcare-seeking behavior, disparities in healthcare access, and social support may also partially explain this association, as women in resource-constrained settings like Bangladesh, may face delays or barriers in accessing timely healthcare, leading to presentation at a later stage of illness with greater symptom burden and greater quality-of-life impairment in this facility-based sample. The findings of our study suggest that, despite the typically self-limiting nature of chikungunya, its acute manifestations impose a negative impact on patients’ functional health and wellbeing. The observed high prevalence of moderate-to-severe pain, functional incapacity, and psychological distress during the acute illness indicates the need for early symptomatic management. The significant association of female sex with worse outcomes indicates the necessity of integrating gender-sensitive approaches into outbreak response and rehabilitation programs. However, the outcome variables, like quality of life or severity of pain was not significantly associated with the laboratory parameters of the participants, most likely due to the limited availability of laboratory data, with sample sizes ranging from 42 to 88 observations across different parameters. Further large scale studies with detailed laboratory evaluation is needed to explore these associations.

From a public health perspective, the resurgence of chikungunya after several years of low transmission signals the re-establishment of viral circulation in Bangladesh, likely facilitated by favorable climatic conditions, vector proliferation, and population mobility [14,37,38]. Considering the fact that the country has experienced recurrent dengue outbreaks in recent years [39–41], the simultaneous circulation of both arboviruses could strain diagnostic and clinical management capacities. Therefore, strengthening differential diagnosis algorithms and vector surveillance systems is essential for early detection and containment. The overlap in clinical presentation between chikungunya and dengue, particularly in early febrile stages, further complicates syndromic diagnosis, which may lead to under-recognition of mixed outbreaks and delayed targeted management. Besides, the health-related quality of life data presented here provide empirical evidence of the human cost of chikungunya outbreaks beyond morbidity and mortality figures. Acute-phase disability, even if transient, can disrupt productivity and livelihood, particularly among working-age adults who constituted the majority of our study participants.

Based on our findings, several recommendations can be made. First, clinical management should prioritize effective pain control, early mobilization, and psychosocial support during the acute phase, particularly for women. Second, routine chikungunya surveillance should incorporate quality-of-life and functional assessments to better quantify disease burden. Third, longitudinal studies are needed to identify predictors of chronic arthralgia and long-term disability. Fourth, comparative studies between chikungunya and dengue could further elucidate shared and distinct determinants of post-viral morbidity, informing integrated arboviral control and rehabilitation strategies.

Our study adds novel multicenter evidence from Bangladesh during the 2025 outbreak by simultaneously quantifying health-related quality of life using EQ-5D-3L and identifying clinical and demographic predictors of impairment at scale. To our knowledge, this is among the first studies in the country to integrate standardized utility-based quality-of-life measurement with clinical severity profiles of chikungunya in a large group of patients across multiple tertiary hospitals. However, several limitations of our study should be acknowledged. First, the facility-based design may introduce selection bias, as patients presenting to healthcare facilities may represent more symptomatic or severe cases, thereby potentially overestimating the degree of quality of life impairment. Second, the cross-sectional nature precludes assessment of symptom persistence or long-term recovery trajectories. Considering the known chronic sequelae of chikungunya, longitudinal follow-up studies are needed to explore post-acute outcomes. Third, the reliance on self-reported symptoms and duration estimates may introduce the possibility of reporting bias. Pain assessment was performed based on VAS, without detailed information on pain duration, temporal pattern, or functional impact, which might limit our ability of deeper interpretation of pain-related disability in these patients. Fourth, although the EQ-5D is a validated generic tool, disease-specific instruments might reflect specific aspects of chikungunya-related disability more precisely. In addition, a Bangladesh-specific EQ-5D TTO value set was not available during the analysis; therefore, we used the Pakistani TTO value set as the closest available regional proxy from South Asia. However, the use of a non-local valuation set might introduce utility estimation bias, as population preferences for different health states may vary across countries due to sociocultural, demographic, and healthcare-system differences, potentially leading to over- or underestimation of quality-of-life impairment. Fifth, laboratory parameters, including inflammatory biomarkers, were not included in the multivariable analyses due to a high level of missingness which limited our ability to explore their association with QoL or disease severity. Sixth, although we screened participants for dengue virus NS1 antigen and IgM antibodies using ELISA to exclude co-infections, early infections may still show overlapping presentation, with possibility of misclassification. Finally, we could not do molecular characterization of circulating viral strains, which limited our ability to assess possible genotype–phenotype correlations and their association with clinical severity.

In summary, majority of the patients infected during the 2025 chikungunya outbreak in Bangladesh presented with classical clinical manifestations but substantial acute-phase disability and impaired quality of life. Pain severity and female sex were significantly associated with poor quality of life, suggesting the importance of targeted symptomatic management and gender-sensitive care. These findings highlight the need for integrated clinical, rehabilitative, and public health responses to mitigate the broader human and societal burden of chikungunya in endemic regions.

## Supporting information

S1 Table Association of laboratory parameters with pain severity. (DOCX)

S2 Table Association of laboratory parameters with EQ-5D index. (DOCX)

S1 File Data. (XLSX)
