# Supplementary material for: Acute-phase clinical profile and quality of life in patients with chikungunya during the 2025 outbreak in Bangladesh: A facility-based cross-sectional study
Source: PLoS Negl Trop Dis. 2026 Aug 26;20(8):e0014599. doi: 10.1371/journal.pntd.0014599 (PMC13577550; doi:10.1371/journal.pntd.0014599)
Supplement: Supplementary file 1 [file pntd.0014599.s001.docx]

**S1 Table:** **Association of laboratory parameters with pain severity**

| **Variables** | **Mild, n (%)** | **Moderate, n (%)** | **Severe, n (%)** | **p-value** |
| --- | --- | --- | --- | --- |
| IgG |  |  |  |  |
| Negative (n=402) | 17 (4.2) | 154 (38.3) | 220 (54.7) | <0.001 |
| Positive (n=40) | 10 (25.0) | 18 (45.0) | 12 (30.0) |  |
| IgM |  |  |  |  |
| Negative (n=140) | 7 (5.0) | 48 (34.3) | 81 (57.9) | 0.263 |
| Positive (n=302) | 20 (6.6) | 124 (41.1) | 151 (50.0) |  |
| PCR |  |  |  |  |
| Negative (n=323) | 18 (5.6) | 128 (39.6) | 172 (53.3) | 0.685 |
| Positive (n=119) | 9 (7.6) | 44 (37.0) | 60 (50.4) |  |
| Platelet count |  |  |  |  |
| ≤100,000 (n=2) | 1 (50.0) | 1 (50.0) | 0 (0.0) | 0.163^†^ |
| >100,000 (n=85) | 5 (5.9) | 62 (72.9) | 11 (12.9) |  |
| Serum ALT |  |  |  |  |
| ≤48 (n=62) | 4 (6.5) | 40 (64.5) | 12 (19.4) | 0.272^†^ |
| >48 (n=11) | 0 (0.0) | 10 (90.9) | 0 (0.0) |  |
| Serum AST |  |  |  |  |
| ≤48 (n=48) | 2 (4.2) | 32 (66.7) | 10 (20.8) | 0.285^†^ |
| >48 (n=3) | 1 (33.3) | 2 (66.7) | 0 (0.0) |  |

^†^Fisher’s exact test
