# Supplementary material for: Acute-phase clinical profile and quality of life in patients with chikungunya during the 2025 outbreak in Bangladesh: A facility-based cross-sectional study
Source: PLoS Negl Trop Dis. 2026 Aug 26;20(8):e0014599. doi: 10.1371/journal.pntd.0014599 (PMC13577550; doi:10.1371/journal.pntd.0014599)
Supplement: Supplementary file 2 [file pntd.0014599.s002.docx]

**S2 Table:** Association of laboratory parameters with EQ-5D index

| **Variables** | **EQ-5D index mean** | **p-value** |
| --- | --- | --- |
| IgG |  |  |
| Negative (n=402) | 0.648 | 0.080 |
| Positive (n=40) | 0.727 |  |
| IgM |  |  |
| Negative (n=140) | 0.629 | 0.261 |
| Positive (n=302) | 0.668 |  |
| PCR |  |  |
| Negative (n=323) | 0.640 | 0.091 |
| Positive (n=119) | 0.696 |  |
| Platelet count |  |  |
| ≤100,000 (n=2) | 0.891 | 0.285 |
| >100,000 (n=85) | 0.683 |  |
| Serum ALT |  |  |
| ≤48 (n=62) | 0.695 | 0.917 |
| >48 (n=11) | 0.685 |  |
| Serum AST |  |  |
| ≤48 (n=48) | 0.671 | 0.858 |
| >48 (n=3) | 0.609 |  |
