# Supplementary material for: Acute-phase clinical profile and quality of life in patients with chikungunya during the 2025 outbreak in Bangladesh: A facility-based cross-sectional study
Source: PLoS Negl Trop Dis. 2026 Aug 26;20(8):e0014599. doi: 10.1371/journal.pntd.0014599 (PMC13577550; doi:10.1371/journal.pntd.0014599)
Supplement: Supplementary file 3 [file pntd.0014599.s005.docx]

**Response to Reviewers**

**Editor Comments:**

The authors must carefully address the concerns raised by the reviewers before submitting a revised version for further consideration.

**Journal Requirements:**

If the reviewer comments include a recommendation to cite specific previously published works, please review and evaluate these publications to determine whether they are relevant and should be cited. There is no requirement to cite these works unless the editor has indicated otherwise.

1) Your manuscript's sections are not in the correct order. Please amend to the following order: Abstract, Introduction, Results, Discussion, and Methods

2) Please upload all main figures as separate Figure files in .tif or .eps format. For more information about how to convert and format your figure files please see our guidelines:

https://journals.plos.org/plosntds/s/figures

3) We have noticed that you have uploaded Supporting Information files, but you have not included a list of legends. Please add a full list of legends for your Supporting Information files after the references list.

***Authors’ response:*** *We sincerely thank the editor for the careful review of our manuscript. In response to the suggestions,*

*1. The manuscript has been revised and the sections have now been reorganized in the required order: Abstract, Introduction, Results, Discussion, and Methods.*

*2. All main figures have been converted and uploaded as separate files in .eps format in accordance with the journal guidelines.*

*3. A list of legends for all Supporting Information files has now been added after the references section in the revised manuscript.*

**Reviewers' Comments:**

**Methods**

-Are the objectives of the study clearly articulated with a clear testable hypothesis stated?

-Is the study design appropriate to address the stated objectives?

-Is the population clearly described and appropriate for the hypothesis being tested?

-Is the sample size sufficient to ensure adequate power to address the hypothesis being tested?

-Were correct statistical analysis used to support conclusions?

-Are there concerns about ethical or regulatory requirements being met?

**Reviewer 1:**

Methods part needs adequate information regarding the details about the kits used for more clarity. Specific comments are as follows;

Line 126-128: Most appropriate references for CHIKV IgM ELSA & RT-PCR may be cited (the cited references 17 &18 deal with diagnostic criteria) may be checked.

Line 157-158: Details about the Dengue NS1 detection for screening may be given.

Line 153-154: Details about the CHIKV IgM ELISA Kit used for screening will be informative.

Line 155-157 More details about the RT-PCR kits used (either in-house optimized or commercially available) The gene of target for RT-PCR amplification (with appropriate reference may be cited) may be given.

As Dengue is a common problem locally, it is worth including information about the circulating CHIKV genotype from human cases at least to a limited sample number in order to confirm the CHIKV etiology during this episode, though RT-PCR amplification was observed. Such information would be useful epidemiologically while correlating with clinical outcome of the strains globally.

***Authors’ response:*** *We thank the reviewer for the constructive comments. Acordingly, we have revised the Methods section to include additional details regarding the diagnostic kits used, including the CHIKV IgM ELISA kit and the Dengue NS1 and IgM/IgG ELISA kits used for screening of co-infections. Regarding the RT-PCR assay, we would like to clarify that the assay was performed using a commercially available multiplex RT-PCR system. As per the manufacturer’s protocol, the specific gene targets were not separately specified in the user documentation; therefore, we are unable to explicitly report individual gene targets.*

*We also acknowledge the reviewer’s important suggestion regarding viral genotype characterization. However, molecular sequencing and genotype determination were not performed in this study, as the RT-PCR was intended for diagnostic confirmation only. We have now explicitly acknowledged this limitation in the revised manuscript.*

**Reviewer 2:**

This study reports the association of female gender and pain severity with poor quality of life in CHIKV patients from Bangladesh during 2025 and provides the clinical picture of the patients.

The title is given as a Sketch of 2025 CHIKV outbreak in Bangladesh but the study describes about the clinical presentations of patients rather than about the outbreak. Hence title needs modification accordingly

***Authors’ response:*** *We sincerely thank the reviewer for the careful evaluation of our manuscript. In response to the reviewer’s comment, the title has been revised as follows: “Acute-phase clinical profile and quality of life in patients with chikungunya during the 2025 outbreak in Bangladesh: A facility-based cross-sectional study.”*

**Reviewer 3:**

(No Response)

**Results**

-Does the analysis presented match the analysis plan?

-Are the results clearly and completely presented?

-Are the figures (Tables, Images) of sufficient quality for clarity?

**Reviewer 1:**

Yes. The results are clearly and completely presented

***Authors’ response:*** *We thank the reviewer for the positive evaluation of our work and encouraging feedback.*

**Reviewer 2:**

The details about persistence of Joint pain is not provided. If the authors can collect details about how many patients had persistent outbreak and the duration of joint pain, it will be a more value addition to the manuscript

Based on the supplementary data provided, I could see that in IgG positive patients, nearly 25% of the patients the severity of the pain was mild where as in only 4% patients had mild pain in IgG negative and this looks like a very interesting and significant observation which has been missed out. The authors are requested further do the analysis based on this laboratory finding and VAS scores in IgG positive and negative patients.

Detailed analysis of different laboratory parameters with severity of pain and quality of life might throw some interesting observations which can be further exploited as prognostic markers of pain severity.

***Authors’ response:*** *We sincerely thank the reviewer for the thoughtful comments and for highlighting important aspects that could further strengthen the manuscript.*

*In response to the first point, we acknowledge that detailed information on persistence and duration of joint pain could improve the findings but unfortunately these information was not collected in this study. This has now been clearly stated as a limitation in the revised manuscript.*

*Regarding the suggested additional analyses, we have explored the associations between IgG status and pain severity, as well as between other laboratory parameters and both pain severity and EQ-5D index, as recommended (Supplementary Table 1 and 2). However, these analyses did not demonstrate statistically significant associations beyond what is already reported, most likely due to the limited availability of laboratory data, with sample sizes ranging from 42 to 88 observations across different parameters.*

**Reviewer 3:**

(No Response)

**Conclusions**

-Are the conclusions supported by the data presented?

-Are the limitations of analysis clearly described?

-Do the authors discuss how these data can be helpful to advance our understanding of the topic under study?

-Is public health relevance addressed?

**Reviewer 1:**

Conclusion supported the data presented. Limitations has been described. Its public health relevance. Yes, authors discussed how these data can be helpful to advance.

***Authors’ response:*** *We sincerely thank the Reviewer for the positive evaluation of our manuscript.*

**Reviewer 2:**

The authors need to discuss about the association of female gender with lower quality of life in the patients. Moreover, based on the new analysis suggested, discussion needs to be taken forward.

***Authors’ response:*** *We thank the Reviewer for the insightful comment regarding the association of female gender with lower quality of life. In response, we have further strengthened the Discussion section to more clearly elaborate on this association.*

*Additionally, as suggested, we conducted the requested analyses; however, these did not demonstrate statistically significant associations beyond those already reported. This has been explicitly stated in the revised manuscript.*

**Reviewer 3:**

(No Response)

**Summary and General Comments**

**Reviewer 1:**

Overall an informative pieces of work. With minor inputs, it may be accepted

***Authors’ response:*** *We sincerely thank the Reviewer for the positive overall assessment and greatly appreciate the constructive guidance for the improvement of our manuscript.*

**Reviewer 2:**

(No Response)

**Reviewer 3:**

This multicenter cross-sectional study of 442 confirmed chikungunya cases from the 2025 Bangladesh outbreak shows high symptom burden with severe pain and marked quality-of-life impairment despite largely normal laboratory parameters. Female sex and pain severity were the main factors associated with lower EQ-5D scores.

Reviewer comments/questions:

• Please justify the rationale for using Pakistani EQ-5D TTO values and discuss potential bias from applying a non-local valuation set.

***Authors’ response:*** *Thanks for the comment. During the analysis, a Bangladesh-specific EQ-5D value set was not available; therefore, the Pakistani value set was used as the closest available regional proxy from South Asia, considering geographical and cultural similarities. The potential for bias arising from the use of a non-local valuation set, including differences in health state preferences across populations, has now been acknowledged and discussed in the Limitations section of the revised manuscript.*

• Laboratory parameters are described but not analytically linked to outcomes; consider assessing their association with QoL or severity.

***Authors’ response:*** *In accordance with this suggestion, we have explored the associations between laboratory parameters and both pain severity and EQ-5D index, as presented in Supplementary Tables 1 and 2. These analyses did not demonstrate statistically significant associations beyond those reported already, most likely due to limited availability of laboratory data, with sample sizes ranging from 42 to 88 observations across different parameters.*

• No inflammatory biomarkers were explored; this limits interpretation of the severe pain and QoL impairment.

***Authors’ response:*** *We thank the reviewer for highlighting this important point. We agree that the absence of inflammatory biomarker data limits the ability to explore potential biological correlates of pain severity and quality-of-life impairment. We have now acknowledged this as a limitation in the revised manuscript.*

• Viral strain differences were not assessed; this is a missed opportunity to relate genotype with clinical severity.

***Authors’ response:*** *We appreciate the reviewer’s insightful comment. We agree that viral genotyping could have provided important epidemiological and clinical insights, particularly regarding potential associations between viral genetic variation and disease severity. However, molecular sequencing and genotypic characterization were beyond the scope of the current study, which was designed primarily for diagnostic confirmation during an ongoing outbreak. We have now acknowledged this as a limitation in the revised manuscript.*

• Pain assessment relies only on VAS; no data on duration, pattern, or functional correlation are provided.

***Authors’ response:*** *We acknowledge that more detailed characterization of pain duration, pattern, and functional impact would have strengthened the analysis. However, unfortunately, such information was not collected in this study. This has now been acknowledged as a limitation in the revised manuscript.*

• Why was convenience sampling used instead of consecutive sampling?

***Authors’ response:*** *Thanks to the reviwer for the generous and insightful comment.*

*Convenience sampling was selected because it was more feasible within the available study duration, workforce, and logistical constraints across the participating hospital as you may know that the project was not supported by any grant. Data collection depended on the availability and willingness of eligible participants during the study period, and it was not always possible to approach every eligible individual consecutively in busy clinical settings. In addition, variations in patient flow, physician workload, and administrative limitations made strict consecutive recruitment difficult to implement uniformly across all sites. Therefore, convenience sampling was considered a practical approach to ensure timely data collection while still capturing participants from the target population. However, we acknowledge that this approach may introduce selection bias and could limit the generalisability of the findings.*

• Were QoL assessments performed at a standardized time point in the illness course?

***Authors’ response:*** *We thank the reviewer for this important clarification. We consider that quality of life assessment was performed at a reasonably standardized point in the clinical course. Specifically, QoL was measured during the acute phase of illness, with a median duration from symptom onset of 5.0 days (IQR 3.0–7.0), and all assessments were completed within 48 hours of hospital admission. As such, although minor variation in symptom duration existed at presentation, the timing of QoL measurement was largely consistent across participants and reflects a comparable stage of acute disease.*

• What were the criteria for hospital admission?

***Authors’ response:*** *We thank the reviewer for raising this point. Hospital admission was determined by the clinical judgment of the attending emergency physicians. Decisions were based on overall symptom severity, functional limitation, and the perceived need for inpatient observation or supportive management. This information has now been stated in the Methods section of the revised manuscript.*

• Were any severe complications (neurological, cardiac) observed?

***Authors’ response:*** *No severe systemic complications, including neurological or cardiac manifestations, were observed among the study participants. This has been added to the Results section.*

• Could differences in healthcare-seeking behavior explain the association with female sex?

***Authors’ response:*** *We agree with this important point and have now further elaborated in the Discussion that differences in healthcare-seeking behavior, including delayed access and variation in presentation patterns among women, may partly explain the observed association with lower quality of life.*

• Were there any cases with thrombocytopenia or transaminitis trends?

***Authors’ response:*** *Thrombocytopenia and transaminitis were uncommon in our participants. Platelet counts below 100,000/µL were observed in two patients, while elevated ALT (>48 U/L) and AST (>48 U/L) were observed in 11 and 3 patients, respectively. These findings have now been added to the Results section.*

• How were dengue co-infections excluded, especially in early illness where overlap is common?

***Authors’ response:*** *To exclude dengue co-infection, all participants were tested using dengue virus NS1 antigen and IgM antibody ELISA assays. This has now been described in the Methods section. We also acknowledge in the Limitations that early clinical overlap between dengue and chikungunya may still result in a small degree of misclassification despite laboratory screening.*

• A forest plot for regression results would improve clarity over tables.

***Authors’ response:*** *We thank the reviewer for this helpful suggestion. We have already included forest plots for the domain-specific regression analyses. However, for the Tobit regression model of the EQ-5D index, we have retained the tabular format, as the relatively wide range of coefficient estimates could reduce interpretability and visual precision in a forest plot format. We believe this balanced approach improves overall clarity while maintaining appropriate statistical presentation.*

• The discussion is largely descriptive; the authors should better compare and contrast their findings with prior studies and provide scientific reasoning for similarities or differences.

***Authors’ response:*** *Thank you for this helpful suggestion. The Discussion section has been revised accordingly to include more detailed comparisons with existing literature and to provide clearer scientific interpretation of similarities and differences across studies.*

• It would be great if the authors can articulate the novelty of this study.

***Authors’ response:*** *We sincerely thank the reviewer for this important suggestion. We have now clarified the novelty of the study in the revised manuscript. In particular, our study provides multicenter evidence from Bangladesh during the 2025 chikungunya outbreak by concurrently assessing health-related quality of life using the EQ-5D-3L instrument and examining its clinical and demographic determinants in a relatively large cohort. To the best of our knowledge, this is among the first studies in the country to integrate utility-based quality-of-life measurement with detailed clinical severity profiling across multiple tertiary care hospitals during an active outbreak, thereby adding both epidemiological and health outcome–oriented insights.*

*We sincerely thank all reviewers for their careful evaluation of our manuscript and for their constructive and insightful comments. Their feedback has been highly valuable in improving the clarity, methodological rigor, and overall quality of the revised manuscript.*
