# Supplementary material for: Acute-phase clinical profile and quality of life in patients with chikungunya during the 2025 outbreak in Bangladesh: A facility-based cross-sectional study
Source: PLoS Negl Trop Dis. 2026 Aug 26;20(8):e0014599. doi: 10.1371/journal.pntd.0014599 (PMC13577550; doi:10.1371/journal.pntd.0014599)
Supplement: Peer review history

# Peer review: Decision Letter 0

**DOI:** 10.1371/journal.pntd.0014599.r001

**Authors:** Esaki M. Shankar, Sujatha Sunil

7 May 2026

Sketch of 2025 Chikungunya Outbreak in Bangladesh: A Multicenter Investigation

PLOS Neglected Tropical Diseases

Dear Dr. Hasan,

Thank you for submitting your manuscript to PLOS Neglected Tropical Diseases. After careful consideration, we feel that it has merit but does not fully meet PLOS Neglected Tropical Diseases's publication criteria as it currently stands. Therefore, we invite you to submit a revised version of the manuscript that addresses the points raised during the review process.

Please submit your revised manuscript within by Jul 06 2026 11:59PM. If you will need more time than this to complete your revisions, please reply to this message or contact the journal office at plosntds@plos.org. When you're ready to submit your revision, log on to https://www.editorialmanager.com/pntd/ and select the 'Submissions Needing Revision' folder to locate your manuscript file.

Please include the following items when submitting your revised manuscript:

* A letter that responds to each point raised by the editor and reviewer(s). You should upload this letter as a separate file labeled 'Response to Reviewers'. This file does not need to include responses to any formatting updates and technical items listed in the 'Journal Requirements' section below.

* A marked-up copy of your manuscript that highlights changes made to the original version. You should upload this as a separate file labeled 'Revised Manuscript with Track Changes'.

* An unmarked version of your revised paper without tracked changes. You should upload this as a separate file labeled 'Manuscript'.

If you would like to make changes to your financial disclosure, competing interests statement, or data availability statement, please make these updates within the submission form at the time of resubmission. Guidelines for resubmitting your figure files are available below the reviewer comments at the end of this letter.

We look forward to receiving your revised manuscript.

Kind regards,

Esaki M. Shankar, PhD, FRCPath

Academic Editor

PLOS Neglected Tropical Diseases

Sujatha Sunil

Section Editor

PLOS Neglected Tropical Diseases

Shaden Kamhawi

co-Editor-in-Chief

PLOS Neglected Tropical Diseases

orcid.org/0000-0003-4304-636XX

Paul Brindley

co-Editor-in-Chief

PLOS Neglected Tropical Diseases

orcid.org/0000-0003-1765-0002

**Additional Editor Comments:**

The authors must carefully address the concerns raised by the reviewers before submitting a revised version for further consideration.

**Journal Requirements:**

If the reviewer comments include a recommendation to cite specific previously published works, please review and evaluate these publications to determine whether they are relevant and should be cited. There is no requirement to cite these works unless the editor has indicated otherwise.

1) Your manuscript's sections are not in the correct order.  Please amend to the following order: Abstract, Introduction, Results, Discussion, and Methods

2) Please upload all main figures as separate Figure files in .tif or .eps format. For more information about how to convert and format your figure files please see our guidelines:

https://journals.plos.org/plosntds/s/figures

3) We have noticed that you have uploaded Supporting Information files, but you have not included a list of legends. Please add a full list of legends for your Supporting Information files after the references list.

**Reviewers' Comments:**

Reviewer's Responses to Questions

**Key Review Criteria Required for Acceptance?**

As you describe the new analyses required for acceptance, please consider the following:

**Methods**

-Are the objectives of the study clearly articulated with a clear testable hypothesis stated?

-Is the study design appropriate to address the stated objectives?

-Is the population clearly described and appropriate for the hypothesis being tested?

-Is the sample size sufficient to ensure adequate power to address the hypothesis being tested?

-Were correct statistical analysis used to support conclusions?

-Are there concerns about ethical or regulatory requirements being met?

Reviewer #1: Methods part needs adequate information regarding the details about the kits used for more clarity. Specific comments are as follows;

Line 126-128: Most appropriate references for CHIKV IgM ELSA & RT-PCR may be cited (the cited references 17 &18 deal with diagnostic criteria) may be checked.

Line 157-158: Details about the Dengue NS1 detection for screening may be given.

Line 153-154: Details about the CHIKV IgM ELISA Kit used for screening will be informative.

Line 155-157 More details about the RT-PCR kits used (either in-house optimised or commercially available) The gene of target for RT-PCR amplification (with appropriate reference may be cited) may be given.

As Dengue is a common problem locally, it is worth including information about the circulating CHIKV genotype from human cases at least to a limited sample number in order to confirm the CHIKV aetiology during this episode, though RT-PCR amplification was observed. Such information would be useful epidemiologically while correlating with clinical outcome of the strains globally.

Reviewer #2: This study reports the association of female gender and pain severity with poor quality of life in CHIKV patients from Bangladesh during 2025 and provides the clinical picture of the patients.

The title is given as a Sketch of 2025 CHIKV outbreak in Bangladesh but the study describes about the clinical presentations of patients rather than about the outbreak. Hence title needs modification accordingly

Reviewer #3: (No Response)

**********

**Results**

-Does the analysis presented match the analysis plan?

-Are the results clearly and completely presented?

-Are the figures (Tables, Images) of sufficient quality for clarity?

Reviewer #1: Yes. The results are clearly and completely presented

Reviewer #2: The details about persistence of Joint pain is not provided. If the authors can collect details about how many patients had persistent outbreak and the duration of joint pain, it will be a more value addition to the manuscript

Based on the supplementary data provided, I could see that in IgG positive patients, nearly 25% of the patients the severity of the pain was mild where as in only 4% patients had mild pain in IgG negative and this looks like a very interesting and significant observation which has been missed out. The authors are requested further do the analysis based on this laboratory finding and VAS scores in IgG positive and negative patients.

Detailed analysis of different laboratory parameters with severity of pain and quality of life might throw some interesting observations which can be further exploited as prognostic markers of pain severity.

Reviewer #3: (No Response)

**********

**Conclusions**

-Are the conclusions supported by the data presented?

-Are the limitations of analysis clearly described?

-Do the authors discuss how these data can be helpful to advance our understanding of the topic under study?

-Is public health relevance addressed?

Reviewer #1: Conclusion supported the data presented. Limitations has been described. Its public health relevance. Yes authors discussed how these data can be helpful to advance.

Reviewer #2: The authors need to discuss about the association of female gender with lower quality of life in the patients. MOreover, based on the new analysis suggested, discussion needs to be taken forward

Reviewer #3: (No Response)

**********

**Editorial and Data Presentation Modifications?**

Use this section for editorial suggestions as well as relatively minor modifications of existing data that would enhance clarity. If the only modifications needed are minor and/or editorial, you may wish to recommend “Minor Revision” or “Accept”.

Reviewer #1: With minor inputs the manuscript can be accepted.

Reviewer #2: (No Response)

Reviewer #3: (No Response)

**********

**Summary and General Comments**

Use this section to provide overall comments, discuss strengths/weaknesses of the study, novelty, significance, general execution and scholarship. You may also include additional comments for the author, including concerns about dual publication, research ethics, or publication ethics. If requesting major revision, please articulate the new experiments that are needed.

Reviewer #1: Overall an informative pieces of work. With minor inputs, it may be accepted

Reviewer #2: (No Response)

Reviewer #3: This multicenter cross-sectional study of 442 confirmed chikungunya cases from the 2025 Bangladesh outbreak shows high symptom burden with severe pain and marked quality-of-life impairment despite largely normal laboratory parameters. Female sex and pain severity were the main factors associated with lower EQ-5D scores.

Reviewer comments/questions:

• Please justify the rationale for using Pakistani EQ-5D TTO values and discuss potential bias from applying a non-local valuation set.

• Laboratory parameters are described but not analytically linked to outcomes; consider assessing their association with QoL or severity.

• No inflammatory biomarkers were explored; this limits interpretation of the severe pain and QoL impairment.

• Viral strain differences were not assessed; this is a missed opportunity to relate genotype with clinical severity.

• Pain assessment relies only on VAS; no data on duration, pattern, or functional correlation are provided.

• Why was convenience sampling used instead of consecutive sampling?

• Were QoL assessments performed at a standardized time point in the illness course?

• What were the criteria for hospital admission?

• Were any severe complications (neurological, cardiac) observed?

• Could differences in healthcare-seeking behavior explain the association with female sex?

• Were there any cases with thrombocytopenia or transaminitis trends?

• How were dengue co-infections excluded, especially in early illness where overlap is common?

• A forest plot for regression results would improve clarity over tables.

• The discussion is largely descriptive; the authors should better compare and contrast their findings with prior studies and provide scientific reasoning for similarities or differences.

• It would be great if the authors can articulate the novelty of this study.

**********

PLOS authors have the option to publish the peer review history of their article (what does this mean?). If published, this will include your full peer review and any attached files.

If you choose “no”, your identity will remain anonymous but your review may still be made public.

**Do you want your identity to be public for this peer review?**  For information about this choice, including consent withdrawal, please see our Privacy Policy.

Reviewer #1: **Yes:** Paramasivan Rajaiah

Reviewer #2: **Yes:** Kalichamy Alagarasu

Reviewer #3: **Yes:** Srinivasu Mudalagiriyappa

**Figure resubmission:**

While revising your submission, we strongly recommend that you use PLOS’s NAAS tool (https://ngplosjournals.pagemajik.ai/artanalysis) to test your figure files. NAAS can convert your figure files to the TIFF file type and meet basic requirements (such as print size, resolution), or provide you with a report on issues that do not meet our requirements and that NAAS cannot fix.

**Reproducibility:**

To enhance the reproducibility of your results, we recommend that authors of applicable studies deposit laboratory protocols in protocols.io, where a protocol can be assigned its own identifier (DOI) such that it can be cited independently in the future. Additionally, PLOS ONE offers an option to publish peer-reviewed clinical study protocols. Read more information on sharing protocols at https://plos.org/protocols?utm_medium=editorial-email&utm_source=authorletters&utm_campaign=protocols

# Author comment: Author response to Decision Letter 1

**DOI:** 10.1371/journal.pntd.0014599.r002

3 Jun 2026

Attachment Submitted filename: 2. Response to Reviewers_JH-11.docx

# Peer review: Decision Letter 1

**DOI:** 10.1371/journal.pntd.0014599.r003

**Authors:** Esaki M. Shankar, Sujatha Sunil

20 Jul 2026

Dear Dr Hasan,

We are pleased to inform you that your manuscript 'Acute-phase clinical profile and quality of life in patients with chikungunya during the 2025 outbreak in Bangladesh: A facility-based cross-sectional study' has been provisionally accepted for publication in PLOS Neglected Tropical Diseases.

Before your manuscript can be formally accepted you will need to complete some formatting changes, which you will receive in a follow up email. A member of our team will be in touch with a set of requests.

Please note that your manuscript will not be scheduled for publication until you have made the required changes, so a swift response is appreciated.

IMPORTANT: The editorial review process is now complete. PLOS will only permit corrections to spelling, formatting or significant scientific errors from this point onwards. Requests for major changes, or any which affect the scientific understanding of your work, will cause delays to the publication date of your manuscript.

Should you, your institution's press office or the journal office choose to press release your paper, you will automatically be opted out of early publication. We ask that you notify us now if you or your institution is planning to press release the article. All press must be co-ordinated with PLOS.

Thank you again for supporting Open Access publishing; we are looking forward to publishing your work in PLOS Neglected Tropical Diseases.

Best regards,

Esaki M. Shankar, PhD, FRCPath

Academic Editor

PLOS Neglected Tropical Diseases

Sujatha Sunil

Section Editor

PLOS Neglected Tropical Diseases

Shaden Kamhawi

co-Editor-in-Chief

PLOS Neglected Tropical Diseases

orcid.org/0000-0003-4304-636XX

Paul Brindley

co-Editor-in-Chief

PLOS Neglected Tropical Diseases

orcid.org/0000-0003-1765-0002

***********************************************************

p.p1 {margin: 0.0px 0.0px 0.0px 0.0px; line-height: 16.0px; font: 14.0px Arial; color: #323333; -webkit-text-stroke: #323333}span.s1 {font-kerning: none

Reviewer's Responses to Questions

**Key Review Criteria Required for Acceptance?**

As you describe the new analyses required for acceptance, please consider the following:

**Methods**

-Are the objectives of the study clearly articulated with a clear testable hypothesis stated?

-Is the study design appropriate to address the stated objectives?

-Is the population clearly described and appropriate for the hypothesis being tested?

-Is the sample size sufficient to ensure adequate power to address the hypothesis being tested?

-Were correct statistical analysis used to support conclusions?

-Are there concerns about ethical or regulatory requirements being met?

Reviewer #2: Yes

Reviewer #3: (No Response)

**********

**Results**

-Does the analysis presented match the analysis plan?

-Are the results clearly and completely presented?

-Are the figures (Tables, Images) of sufficient quality for clarity?

Reviewer #2: Yes

Reviewer #3: (No Response)

**********

**Conclusions**

-Are the conclusions supported by the data presented?

-Are the limitations of analysis clearly described?

-Do the authors discuss how these data can be helpful to advance our understanding of the topic under study?

-Is public health relevance addressed?

Reviewer #2: Yes

Reviewer #3: (No Response)

**********

**Editorial and Data Presentation Modifications?**

Use this section for editorial suggestions as well as relatively minor modifications of existing data that would enhance clarity. If the only modifications needed are minor and/or editorial, you may wish to recommend “Minor Revision” or “Accept”.

Reviewer #2: (No Response)

Reviewer #3: (No Response)

**********

**Summary and General Comments**

Use this section to provide overall comments, discuss strengths/weaknesses of the study, novelty, significance, general execution and scholarship. You may also include additional comments for the author, including concerns about dual publication, research ethics, or publication ethics. If requesting major revision, please articulate the new experiments that are needed.

Reviewer #2: I am satisfied with the revision and response

Reviewer #3: The authors have satisfactorily addressed the comments and suggestions raised during the review process. I believe the manuscript has improved significantly and recommend that it be accepted for publication in the journal.

**********

PLOS authors have the option to publish the peer review history of their article (what does this mean?). If published, this will include your full peer review and any attached files.

If you choose “no”, your identity will remain anonymous but your review may still be made public.

**Do you want your identity to be public for this peer review?**  For information about this choice, including consent withdrawal, please see our Privacy Policy.

Reviewer #2: **Yes:** Kalichamy Alagarasu

Reviewer #3: No

# Editor report: Acceptance letter

**DOI:** 10.1371/journal.pntd.0014599.r004

**Authors:** Esaki M. Shankar, Sujatha Sunil

Dear Dr Hasan,

We are delighted to inform you that your manuscript, "Acute-phase clinical profile and quality of life in patients with chikungunya during the 2025 outbreak in Bangladesh: A facility-based cross-sectional study," has been formally accepted for publication in PLOS Neglected Tropical Diseases.

We have now passed your article onto the PLOS Production Department who will complete the rest of the publication process. All authors will receive a confirmation email upon publication.

The corresponding author will soon be receiving a typeset proof for review, to ensure errors have not been introduced during production. Please review the PDF proof of your manuscript carefully, as this is the last chance to correct any scientific or type-setting errors. Please note that major changes, or those which affect the scientific understanding of the work, will likely cause delays to the publication date of your manuscript. Note: Proofs for Front Matter articles (Editorial, Viewpoint, Symposium, Review, etc...) are generated on a different schedule and may not be made available as quickly.

Soon after your final files are uploaded, the early version of your manuscript will be published online unless you opted out of this process. The date of the early version will be your article's publication date. The final article will be published to the same URL, and all versions of the paper will be accessible to readers.

For Research Articles, you will receive an invoice from PLOS for your publication fee after your manuscript has reached the completed accept phase. If you receive an email requesting payment before acceptance or for any other service, this may be a phishing scheme. Learn how to identify phishing emails and protect your accounts at https://explore.plos.org/phishing.

Thank you again for supporting open-access publishing; we are looking forward to publishing your work in PLOS Neglected Tropical Diseases.

Best regards,

Shaden Kamhawi

co-Editor-in-Chief

PLOS Neglected Tropical Diseases

Paul Brindley

co-Editor-in-Chief

PLOS Neglected Tropical Diseases
